# An amino acid-based oral rehydration solution (AA-ORS) enhanced intestinal epithelial proliferation in mice exposed to radiation

**DOI:** 10.1038/srep37220

**Published:** 2016-11-23

**Authors:** Liangjie Yin, Reshu Gupta, Lauren Vaught, Astrid Grosche, Paul Okunieff, Sadasivan Vidyasagar

**Affiliations:** 1 Department of Radiation Oncology, University of Florida Health Cancer Center, Cancer and Genetics Research Complex, 2033 Mowry Road, Box 103633, Gainesville, FL 32610, USA

## Abstract

Destruction of clonogenic cells in the crypt following irradiation are thought to cause altered gastrointestinal function. Previously, we found that an amino acid-based oral rehydration solution (AA-ORS) improved gastrointestinal function in irradiated mice. However, the exact mechanisms were unknown. Electrophysiology, immunohistochemistry, qPCR, and Western blot analysis were used to determine that AA-ORS increased proliferation, maturation, and differentiation and improved electrolyte and nutrient absorption in irradiated mice. A single-hit, multi-target crypt survival curve showed a significant increase in crypt progenitors in irradiated mice treated with AA-ORS for six days (8.8 ± 0.4) compared to the saline-treated group (6.1 ± 0.3; P < 0.001) without a change in D_0_ (4.8 ± 0.1 Gy). The D_q_ values increased from 8.8 ± 0.4 Gy to 10.5 ± 0.5 Gy with AA-ORS treatment (P < 0.01), indicating an increased radiation tolerance of 1.7 Gy. We also found that AA-ORS treatment (1) increased Lgr5^+^, without altering Bmi1 positive cells; (2) increased levels of proliferation markers (Ki-67, p-Erk, p-Akt and PCNA); (3) decreased apoptosis markers, such as cleaved caspase-3 and Bcl-2; and (4) increased expression and protein levels of NHE3 and SGLT1 in the brush border membrane. This study shows that AA-ORS increased villus height and improved electrolyte and nutrient absorption.

Gastrointestinal (GI) toxicity manifests itself in the first week following accidental and therapeutic radiation exposure and is the most significant dose-limiting factor in the clinical course of radiotherapy^1^,^2^,^3^,^4^,^5^,^6^. Currently, there is no FDA-approved agent for its prevention or treatment^7^,^8^,^9^. Moderate to high doses of radiation result in the destruction of cells with clonogenic potential, which are essential for the continuous replacement of cells that are shed from the top of the villi during the normal proliferation, maturation, and differentiation process^10^. Differentiated villus cells are involved in fluid absorption secondary to sodium (Na^+^), chloride (Cl^−^), and nutrient absorption, whereas the less differentiated, immature epithelial cells located in the crypt are predominantly involved in Cl^−^ secretion and fluid loss. The lack of absorptive villus epithelial cells leads to a malabsorptive state in which unabsorbed nutrients, electrolytes, and water are dumped into the distal segments of the GI tract, resulting in nausea, vomiting, and diarrhea. Stem cell-mediated repopulation of villus cells through proliferation of *in situ* cells are thought to be responsible for recovery from acute irradiation effects at the tissue level.^11^,^12^,^13^ Circulation of progenitor cells from distant sites migrating into tissues may help in the recovery from radiation toxicity, however, recent report from Brian J. Leibowitz *et al*. has shown that the bone marrow derived cells may not have a significant role^14^.

Human intestinal epithelial cells are generated from a fixed population of stem cells functionally situated in the lower portion of the intestinal crypts, including fast cycling crypt base columnar cells (CBCs) and more quiescent “+4” cells above Paneth cells in mice^15^,^16^,^17^. These stem cells give rise to absorptive enterocytes, mucus cells, Paneth cells, and enteroendocrine cells^18^. The differentiation of each cell type occurs when cells either move upwards into the villus (absorptive, mucus, and endocrine cells) or concentrate downwards at the bottom of the crypt (Paneth cells)^18^. The multiple mechanisms responsible for these complex events are not fully understood.

Using electrophysiological techniques, we have shown that radiation-induced Cl^−^ secretion can occur at radiation doses that are too low to cause obvious histopathological changes^19^. Radiation-induced enteric dysfunction was characterized by: (**1**) increased Cl^−^ secretion that was responsible for increased fluid secretion; (**2**) decreased absorption of Na^+^, which led to decreased fluid absorption; and (**3**) increased paracellular permeability that resulted in increased translocation of luminal antigenic substances into the systemic compartment, generating a local and systemic immune response. Increased permeation of luminal contents into the systemic compartment increased plasma endotoxin and proinflammatory cytokines (e.g., IL1β)^20^.

As outlined in a previous study, we developed an amino acid-based oral rehydration solution (AA-ORS)^20^. Particular amino acids were chosen based on our findings in intestinal tissues from irradiated mice that the selected amino acids: (**1**) increased Na^+^ absorption via amino acids-coupled Na^+^ absorption; (**2**) did not stimulate Cl^−^ secretion and, therefore, fluid secretion; and (**3**) decreased paracellular permeability or tightening of the mucosal barrier. Treatment with AA-ORS for a period of 14 days improved electrolyte absorption, decreased paracellular permeability as well as plasma endotoxin and proinflammatory cytokine levels, better preserved body weight, and improved survival in mice exposed to an otherwise lethal dose of total-body irradiation (8.5 Gy TBI)^20^. Subsequent studies in our laboratory showed that these improvements occurred as early as 7 days after AA-ORS treatment; however, the exact mechanisms for these effects are unknown. The top 5 amino acids were selected from the original list of amino acids and used in the present study; threonine (1.0 grams/litre), valine (1.2 grams/litre), serine (1.1 grams/litre), tyrosine (0.2 grams/litre), and tryptophan (1.6 grams/litre). These select amino acids are now available in enterade^®^ (Entrinsic Health Solutions, LLC, Norwood, MA), a commercial product that is comprised of a mixture of electrolytes and amino acids at pH 4.2, an osmolarity of 232 mOsms, and flavoring agents. This study used a similar mixture of electrolytes and amino acids, but did not include flavoring agents.

In this study, we explored the mechanism that is responsible for better absorption of electrolytes and nutrients with AA-ORS following radiation exposure. We hypothesized that increased villus height induced by our AA-ORS is involved in increased electrolyte and nutrient absorption following irradiation. To study how AA-ORS increases the absorptive surface area by increasing villus height, we investigated increased proliferation and the related signaling pathway both in the presence and absence of AA-ORS following radiation exposure. We found that AA-ORS increased Lgr5^+^ stem cells; increased proliferation markers, such as p-Erk; and decreased apoptosis markers, such as cleaved caspase-3. This study shows that *in situ* stem cells and the intestinal epithelial cell proliferation induced by AA-ORS increased electrolyte and nutrient absorption by increasing villus height that comprised of mature, differentiated epithelial cells.

## Results

### AA-ORS increased crypt count and villus length in intestinal tissues from irradiated mice

**Figure 1:**
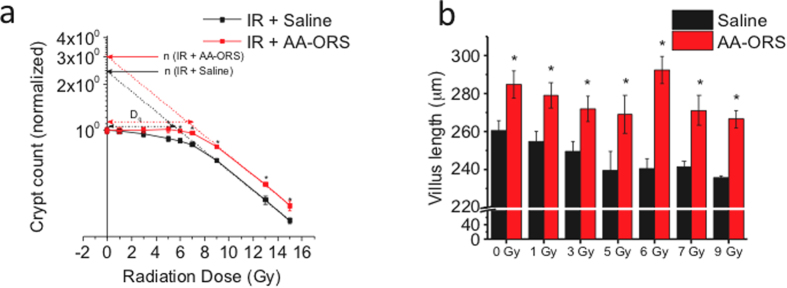
AA-ORS increased crypt count & villus length following irradiation. Normal saline (saline) was used as a control; saline and AA-ORS were given by gastric gavage. 6 mice per radiation group (0, 1, 3, 5, 6, 7, 9, 13 and 15 Gy) with and without treatment. (**a**) Semi-log survival curve showing the effect of AA-ORS on crypt count. AA-ORS shifted the graph to the left. The crypt survival curve was modeled using a single-hit, multi-target cell survival model to assess the biological effect. The probability of survival of the mitotic cells in the crypt following radiation was calculated using the equation [S = 1-(1-e^-D/D_0_)^n^. S represents the fraction of mitotic cells in the crypts that survived in each of the radiation dose, D represents radiation dose; D_0_, a measure of the intrinsic radiation resistance of the crypt reproductive units. Dq values for saline treated mice and AA-ORS treated mice are represented by black arrow and red arrow respectively. Dq is calculated from the formula Dq = D_0_ In n. Without constraining constant cell sensitivity, the N values were 10.4 ± 0.2 and 5.3 ± 0.1 (P < 0.001), indicating a near doubling of progenitor units per circumference from a control. When a constant D_0_ (4.8 ± 0.1 Gy) was constrained, the difference remained significant at 8.8 ± 0.4 to 6.1 ± 0.3 (P < 0.001). (**b**) Shows the height of villus following treatment using saline and AA-ORS in irradiated mice. Significant increase in villus height with AA-ORS treated mice compared to mice receiving saline as treatment. Crypts per circumference were counted, and villus length was measured from 10 sections obtained from the ileum. Data are shown as the mean ± S.E.M. for 6 mice per group. *Indicates statistically significant difference (P < 0.01). Normal saline (saline) was used as control and both saline and AA-ORS was given by gastric gavage.

The intestinal tissues of irradiated mice (5–15 Gy) treated with AA-ORS exhibited a significant increase in crypt count per circumference (N) (Fig. 1a). As crypts are formed by regenerative units, these results indicate an increased number of progenitor epithelial cells. Similarly, villus length significantly increased across all radiation doses with AA-ORS treatment when compared to saline treatment (Fig. 1b). This is consistent with increased progenitor proliferation and/or longer survival of epithelial cells before natural sloughing. The quasi-threshold dose (D_q_) values, a composite measure of crypt tolerance of radiation, were 10.5 ± 0.5 Gy for AA-ORS-treated mice and 8.8 ± 0.4 Gy for saline-treated mice (P < 0.01). The AA-ORS group, as compared to the saline group, had a “broad shoulder” in the low radiation dose region, suggesting an increased number of progenitors per bowel circumference (less senescence) (Fig. 1a). As expected, the terminal portion followed an exponential relationship. All subsequent studies were undertaken in 0 and 5 Gy irradiated mice, since 5 Gy was the lowest radiation before the exponential loss of crypts occurred and because our previous studies have shown that peak anion secretion occurs at 5 Gy without obvious histopathological changes^21^.

### AA-ORS promotes intestinal epithelial cell migration

With the continuous renewal of villus epithelial cells, there is also a continuous migration of cells along the crypt-villus axis^22^. Cell proliferation was studied using EdU incorporation.

**Figure 2:**
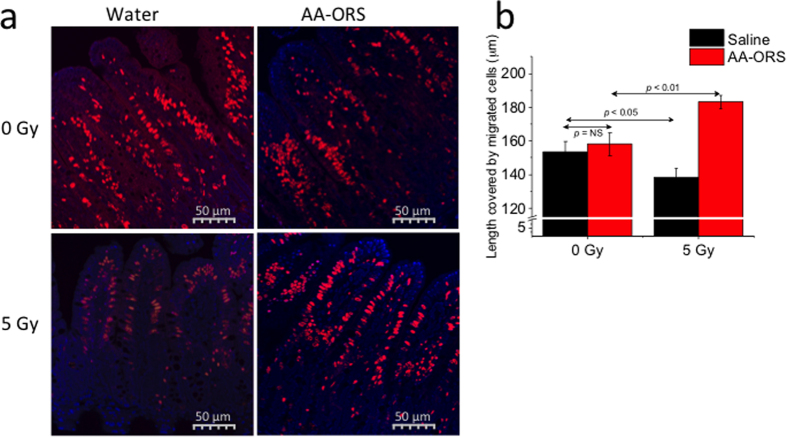
(**a**) Confococal microscopy of longitudinal ileal section with prominently stained epithelial cells along the villus length. Paraffin embedded tissues at 5-μm thickness were used. Cell nuclei were stained with DAPI (blue) and EdU^+^ epithelial cells stained red. Image Pro Plus software was used for measurements of distance migrated by the EdU^+^ cells along the villus height. Bar – 100 μm. Cells were scored per entire crypt and villus unit. At least 60 crypts and corresponding villi were analyzed per mouse. EdU-labeled cells were normalized to the total cell number per crypt or villus. Minimum of five well-oriented villi were counted per tissue section and the results were averaged. EdU^+^ cells were seen all the way to the tip of the villus in 5 Gy irradiated tissues but not in AA-ORS-treated mice. (**b**) EdU^+^ cell migration distance measured at 72 hours. The 5 Gy irradiated saline-treated mice showed significant decrease in cell migration distance (black bar) compared to 0 Gy. AA-ORS had increased migration distance when compared to irradiated saline treated mice. Values are means ± S.E.M. for 6 mice per group.

We found that cells migrated from the crypt base to the villus tip in ~72 hours and therefore in all subsequent studies, EdU was injected 72 hours prior to harvesting the tissues. Ileal sections from 0 Gy mice showed that EdU-incorporated cells reached the villus tip at different times (75 ± 0.9 hours; n = 10 mice). AA-ORS treatment did not lead to a significant difference in the length covered by the migrated cells in 0 Gy mice (153.5 ± 6 μm vs 158.1 ± 7 μm; n = 10 mice). However, AA-ORS treatment led to a significant difference in 5 Gy irradiated mice (158.1 ± 7 μm vs 183.1 ± 4 μm; P < 0.01, n = 10 mice) (Fig. 2a,b). These studies show that AA-ORS increases proliferation and is responsible for the length covered.

### AA-ORS increased Na^+^ and Cl^−^ absorption

**Figure 3:**
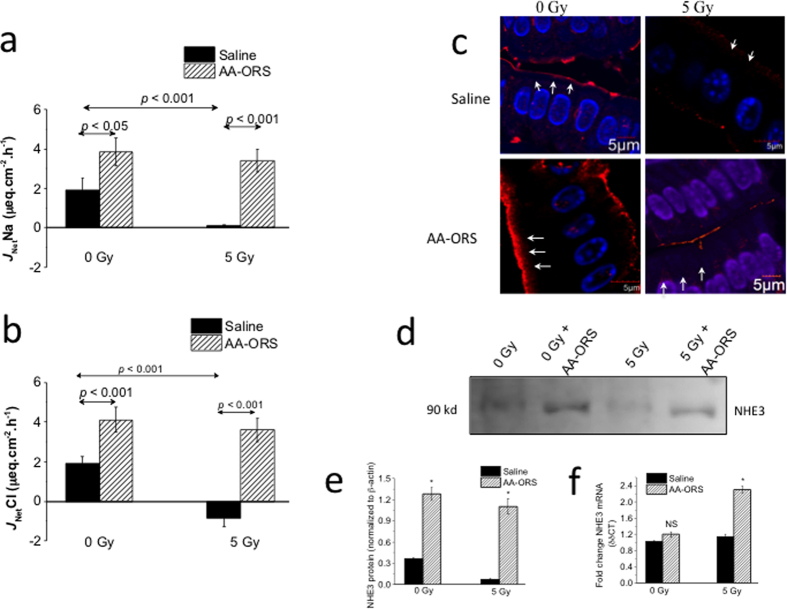
(**a**,**b**) Ussing chamber flux studies using ^22^Na and ^36^Cl showing the effect of AA-ORS on Na^+^ and Cl^−^ absorption. AA-ORS increased net Na^+^ (*J*_net_Na) and Cl^−^ (*J*_net_Cl) absorption in 0 Gy and 5 Gy irradiated tissues (n = 8). (**c**) Immunohistochemistry showing a magnified view of NHE3 expression (red) along the brush border membrane (BBM) of villus epithelial cells (white arrows). Paraffin embedded tissues at 5-μm thickness were used. Cell nuclei were stained with DAPI (blue). A minimum of five well-oriented villi were used. (**d**) Western blot analysis for NHE3 protein, (**e**) graphical representation of NHE3 protein density in intestinal tissues from mice treated with saline (black bars) or AA-ORS (hatched bars) following 0 or 5 Gy irradiation. Immunoblots were repeated four times. Values are means ± S.E.M. from n = 4, *indicates statistically significant difference (P < 0.05) from saline treated animals. (**f**) NHE3 transcript levels in intestinal tissues from mice treated with saline (black bars) or AA-ORS (hatched bars) following 0 or 5 Gy irradiation. Values are means ± S.E.M. from n = 6, *indicates statistically significant difference (P < 0.05) from saline treated animals. Saline or AA-ORS was given for 6 days.

To determine if the increased villus height resulted in functionally mature and differentiated villus epithelial cells, isotope flux studies were undertaken to determine Na^+^ and Cl^−^ absorption. Non-irradiated mice had a net Na^+^ absorption (*J*_Net_Na) of 1.9 ± 0.6 μeq. cm^2^. h^−1^ and a net Cl^−^ absorption (*J*_Net_Cl) of 1.9 ± 0.4 μeq. cm^2^. h^−1^ (Fig. 3a,b). The ileum of 5 Gy irradiated mice had a decrease in *J*_Net_Na (1.9 ± 0.6 μeq. cm^2^. h^−1^ vs 0.1 ± 0.0 μeq. cm^2^. h^−1^; P < 0.001, n = 8) and *J*_Net_Cl (1.9 ± 0.4 μeq. cm^2^. h^−1^ vs −0.9 ± 0.4 μeq. cm^2^. h^−1^; P < 0.001, n = 8). AA-ORS treatment led to a significant increase in net Na^+^ absorption in the ileum of 0 Gy (3.9 ± 0.7 μeq. cm^2^. h^−1^; P < 0.05, n = 8) and 5 Gy (3.4 ± 0.7 μeq. cm^2^. h^−1^; P < 0.001, n = 8) mice (Fig. 3a). Similarly, AA-ORS treatment led to increased Cl^−^ absorption in the ileum of 0 Gy (4.1 ± 0.6 μeq. cm^2^. h^−1^; P < 0.05, n = 8) and 5 Gy (3.6 ± 0.6 μeq. cm^2^. h^−1^; P < 0.001, n = 8) mice (Fig. 3b).

Villus cells from 5 Gy irradiated animals showed little or no NHE3 expression along the brush border membrane when compared to 0 Gy (Fig. 3c). Treatment with AA-ORS increased NHE3 protein expression along the border region in epithelial cells from 0 Gy and 5 Gy irradiated mice (Fig. 3c). Western blot analysis showed increased NHE3 protein levels in the intestinal tissues of AA-ORS-treated mice irradiated at 0 Gy (3.5-fold) and 5 Gy (15.5-fold) compared to saline-treated irradiated mice (Fig. 3d,e). These studies suggest an increase in NHE3 protein in the brush border membrane of the villus epithelial cells. To determine if the increase in NHE3 protein resulted from an increase in NHE3 mRNA, its levels in intestinal tissues were determined using qPCR (Fig. 3f). Unlike NHE3 protein levels, NHE3 mRNA levels were only significantly different at 5 Gy when compared to saline-treated 5 Gy irradiated mice. Protein levels did not correlate well with the changes in NHE3 mRNA levels and similar observation has been reported previously^23^.

### AA-ORS increased glucose-stimulated Na^+^ absorption

**Figure 4:**
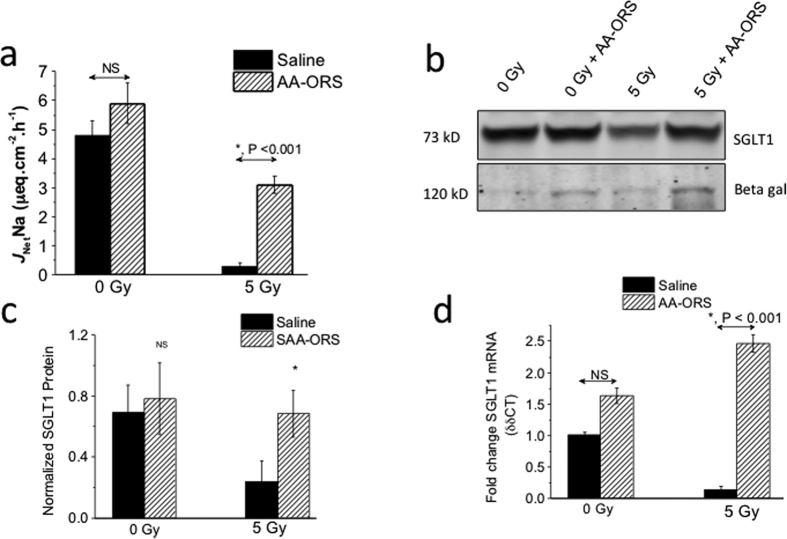
Glucose-stimulated sodium absorption and SGLT1 protein levels. (**a**) Ussing chamber flux studies using ^22^Na showing the effect of AA-ORS on glucose coupled Na absorption. AA-ORS treatment increased *J*_net_Na absorption in 5 Gy irradiated tissues (n = 8). (**b**) Western blot analysis for SGLT1 protein and beta-galactosidase, showed increased protein levels with AA-ORS treatment in villus cells from 0 Gy and 5 Gy mice. Immunoblots were repeated four times. (**c**) Normalized SGLT1 protein levels for western analysis. Significant difference in SGLT1 protein levels was observed in 5 Gy irradiated mice treated with AA-ORS when compared to 5 Gy mice. (**d**) SGLT1 transcript levels in intestinal tissues from mice treated with saline (black bars) or AA-ORS (hatched bars) following 0 or 5 Gy irradiation. Values are means ± S.E.M. from n = 6, *indicates statistically significant difference (P < 0.05) from saline treated animals. Saline or AA-ORS was given for 6 days.

To determine if the AA-ORS-induced increase in villus height resulted in improved glucose absorption, we assessed glucose-stimulated Na^+^ absorption using ^22^Na flux studies. Ileal tissues from 5 Gy irradiated mice showed a significant reduction in glucose-stimulated *J*_Net_Na (4.8 ± 0.5 μeq. cm^2^. h^−1^ vs 0.3 ± 0.1 μeq. cm^2^. h^−1^; P < 0.001, n = 6). Ileal tissues from AA-ORS-treated 5 Gy irradiated mice showed a significant increase in glucose-stimulated *J*_Net_Na (0.3 ± 0.1 μeq. cm^2^. h^−1^ vs 3.1 ± 0.3 μeq. cm^2^. h^−1^; P < 0.001, n = 6), whereas 0 Gy irradiated mice did not exhibit this increase (4.8 ± 0.5 μeq. cm^2^. h^−1^ vs 5.9 ± 0.7 μeq. cm^2^. h^−1^; P = ns, n = 6) (Fig. 4a). AA-ORS treatment enhanced SGLT1 protein levels and at its transcription levels in 0 Gy and 5 Gy irradiated mice compared to saline-treated mice (Fig. 4b–d). These studies suggest that AA-ORS-induced increase in villi heights are functional, by showing that electrolyte absorptive capacity during inter-digestive phase (NHE3-mediated Na^+^ absorption) and digestive phase (glucose-stimulated Na^+^ absorption) are increased, both of which are a function of mature and differentiated villus epithelial cells.

Beta-galactosidase (lactose protein) levels were measured in isolated villus cells using Western blot analysis. Primarily, beta-galactosidase expression occurs in mature and differentiated villus epithelial cells. AA-ORS treatment increased beta-galactosidase protein levels in villus cells in 0 Gy and 5 Gy irradiated mice (Fig. 4b).

### Effect of AA-ORS on intestinal stem cells and proliferation markers

**Figure 5:**
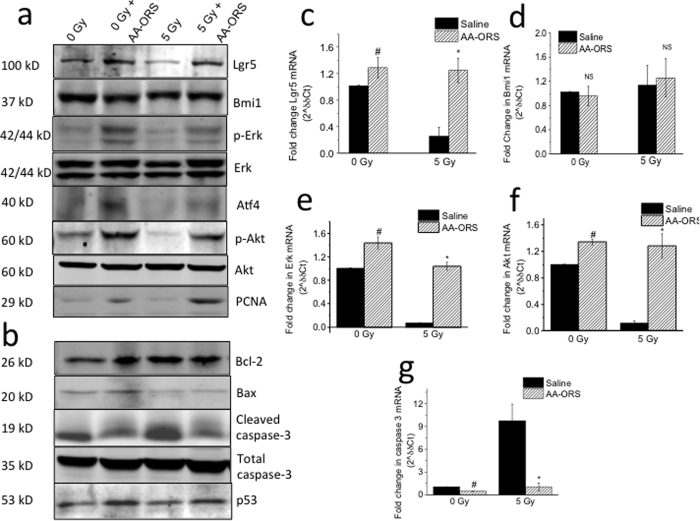
Protein levels and mRNA expression of Lgr5, Bmi1, p-Akt, Akt, p-Erk, Erk in villus epithelial cells from mice treated with saline and AA-ORS following 0 and 5 Gy irradiation. Immunoblots were repeated four times and q-PCR were repeated 6 times. (**a**) Western blot analysis for stem cell and proliferation markers (Lgr5, Bmi1, p-Akt, Akt, p-Erk, Erk and PCNA). The protein band of interest was normalized to the total amount of protein in each lane using Coomassie blue stain. (**b**) Western blot analysis for apoptotic proteins (Bcl2, Bax, cleaved caspase-3, caspase-3 and p53). (**c**) Lgr5 mRNA levels in mice treated with saline or AA-ORS and 0 Gy or 5 Gy radiation. (**d**) Changes in Bmi1 mRNA levels in mice treated with saline or AA-ORS and 0 Gy or 5 Gy. (**e**) Changes in Erk mRNA levels in mice treated with saline or AA-ORS treatment and 0 Gy or 5 Gy. (**f**) Changes in Akt mRNA levels in mice treated with saline or AA-ORS treatment and 0 Gy or 5 Gy. (**g**) mRNA expression for caspase-3. Values are means ± S.E.M from n = 6 different mice repeated in triplicates. ^#^P < 0.05 and *P < 0.001 compared with saline control.

To determine if an increase in stem cell number and proliferation was responsible for the increased villus height observed with AA-ORS, we studied the effect of AA-ORS on markers for stem cells and proliferation. Irradiation resulted in a significant decrease in Lgr5 protein levels (Fig. 5a and Supplementary Fig. 1a). AA-ORS increased Lgr5 protein levels in 0 Gy and 5 Gy irradiated mice when compared to saline-treated control groups. However, intestinal tissues from 5 Gy irradiated mice showed no significant change in Bmi1 protein levels when compared to 0 Gy. Similarly, AA-ORS did not change Bmi1 protein levels in 0 Gy and 5 Gy irradiated mice (Fig. 5a and Supplementary Fig. 1b). Lgr5 transcript levels, but not Bmi1 levels, significantly increased in AA-ORS-treated 0 Gy and 5 Gy mice (Fig. 5c,d).

Western blot analysis of Erk1/2 and p-Erk1/2 were studied using the whole cell fraction to assess the effect of radiation and AA-ORS on proliferation. Erk1/2 and Akt are phosphorylated when activated. Western blot analysis showed a significant difference in p-Erk protein levels at 0 Gy and 5 Gy (Fig. 5a and Supplementary Fig. 1c). Total Erk protein levels were not significantly different in the AA-ORS-treated and saline-treated mice at 0 Gy or 5 Gy. Similarly, with Erk, 0 and 5 Gy mice treated with AA-ORS did not exhibit significant differences in Akt levels, whereas they did exhibit an increase in p-Akt protein levels when compared to the corresponding saline-treated irradiated groups. Intestinal tissues from 5 Gy mice showed a significant decrease in p-Akt when compared to 0 Gy mice (Supplementary Fig. 1d,e). These studies suggest an increased phosphorylation level of the protein with AA-ORS treatment without a change in total protein expression. Since the effect of MAPK is dependent on its downstream effector the activating transcription factor 4 (Atf4), the Atf4 protein levels were measured using western blot analysis^24^. 5 Gy irradiation reduced protein levels of Atf4, but AA-ORS treatment increased Atf4 protein levels in 0 Gy and 5 Gy irradiated mice. To determine the transcript levels of Erk, and Akt, qPCR studies were undertaken in epithelial cells isolated from 0 Gy and 5 Gy irradiated tissues both in the absence and presence of treatment. Erk and Akt mRNA levels increased with AA-ORS treatment in 0 Gy and 5 Gy irradiated mice and paralleled changes in p-Erk and p-Akt protein levels (Fig. 5e,f). Early changes in normal cell proliferation within the intestinal tract serve as an indication of deviation from normal gastrointestinal function. Changes in the expression of proliferating cell nuclear antigen (PCNA), a 36 kD protein is recognized as one such marker for changes in the gut. AA-ORS increased PCNA protein levels in 0 Gy and 5 Gy irradiated mice (Fig. 5a and Supplementary Fig. 2a).

We also studied the effect of AA-ORS on B-cell lymphoma-2 protein (Bcl-2), a downstream target for Erk1/2. Bcl-2 prevents cell death rather than promoting cell proliferation by regulating the expression levels of the pro-apoptotic Bcl-2 associated X-protein (Bax) in the intrinsic caspase cascade^25^. Bcl-2 levels increased with AA-ORS treatment in 0 Gy mice. Irradiation resulted in significant increase in Bcl-2 protein levels. Treatment using AA-ORS did not show further increase in Bcl2 protein levels in 5 Gy irradiated mice (Fig. 5b and Supplementary Fig. 2b). Increased Bcl-2 protein levels with irradiation may suggest a protective mechanism to prevent apoptosis. However, western blot analysis using Bax specific antibodies did not show a significant difference in protein levels (Fig. 5b and Supplementary Fig. 2c). The studies agree with previous observations that interventions targeting Bcl-2 not necessarily change protein levels of Bax^26^.

Activation of caspase-3 results in the formation of a 19 kD cleaved caspase-3. Cleaved caspase-3 increases with apoptosis. Western blot analysis showed no significant difference in total caspase-3 following irradiation and with treatment when compared to the control. Intestinal tissues from 5 Gy mice showed a significant increase in cleaved caspase-3 when compared to tissues from the 0 Gy mice. However, cleaved capase-3 decreased with AA-ORS when compared to the corresponding irradiation controls (Fig. 5b and Supplementary Fig. 1f). Caspase-3 mRNA levels measured using qPCR showed a significant increase in caspase-3 transcript levels following 5 Gy irradiation when compared to 0 Gy. Treatment using AA-ORS resulted in a significant decrease in caspase-3 transcript levels in intestinal tissues from 0 Gy and 5 Gy irradiated mice (Fig. 5g).

Western blot analysis for p53 protein levels showed no significant difference in the p53 (a tumor suppressor protein) level in intestinal tissues from 0 Gy and 5 Gy mice. AA-ORS showed a small but consistent increase in p53 protein levels (Fig. 5b). These studies suggest that the proliferative effect associated with AA-ORS may not be associated with tumorigenesis.

### Immunohistochemistry

**Figure 6:**
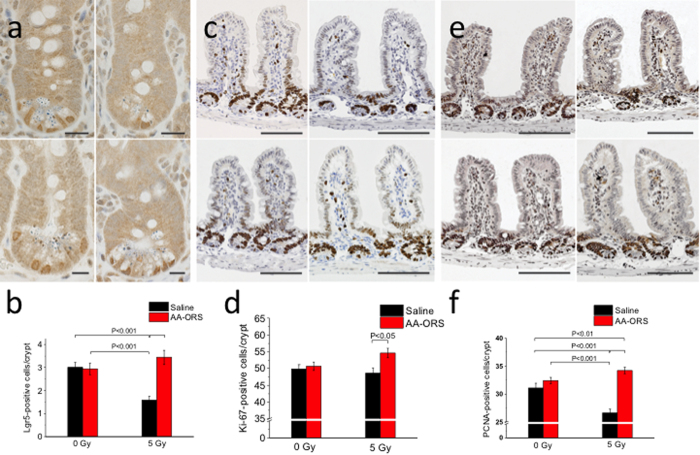
Representative microphotographs of the distribution of Lgr5^+^, Ki-67^+^ and PCNA^+^ cells within ileal mucosa of 0 Gy (left) and 5 Gy (right) after treatment with saline (top) or AA-ORS (bottom). (**a**) Immunostaining for Lgr5: Lgr5^+^ cells were seen in the lower 1/3^rd^ of the crypt. Mice irradiated with 5 Gy resulted in a significant decline of Lgr5^+^ stem cells in ileal crypts, and AA-ORS increased Lgr5^+^ stem cells. Scale bars represent 25 μm (**b**) Mean number of Lgr5^+^ cells expressed in crypt. Error bars indicate S.E.M. (**c**) Immunostaining for Ki-67: The number of Ki-67-expressing cells, a proliferation marker, showed no significant difference in 0 Gy radiated mice treated with AA-ORS when compared to saline-treated groups. 5 Gy irradiated mice showed significant increase in Ki-67^+^ cells with AA-ORS treatment. Scale bars represent 100 μm. (**d**) Mean number of Ki-67 expressing cells in crypt and/or villus cells. Error bars indicate S.E.M. (**e**) Immunostaining for PCNA: The number and distribution of PCNA^+^ cells. PCNA^+^ cells were reduced in mice after 5 Gy radiation, but increased with AA-ORS treatment. Scale bars represent 100 μm. (**f**) Mean number of PCNA expressing cells in crypt and/or villus cells. Error bars indicate S.E.M.

Avidin-biotin detection method using a polyclonal antibody against Lgr5^+^, intestinal stem cells showed a decrease in Lgr5^+^ cells with radiation (3.0 ± 0.2 vs 1.6 ± 0.2; P < 0.001, n = 50 crypts). Treatment using AA-ORS did not show a significant difference in Lgr5^+^ in 0 Gy irradiated mice. However, in 5 Gy irradiated mice AA-ORS increased Lgr5^+^ cells (1.6 ± 0.2 vs 3.4 ± 0.3; P < 0.001, n = 50 crypts) (Fig. 6a & b).

Ki-67 protein, a cellular marker for proliferation is present in the cell during all active phases of cells cycle (G_1_, S, G_2_, and mitosis), but absent from resting cells (G_0_). Ileal sections showed Ki-67 expression along the crypt except its lower pole and the expression extended into the lower 1/3^rd^ of the villi. There was no significant difference in Ki-67 expression with radiation, however, treatment using AA-ORS resulted in significant increase in Ki-67 expressed cells (50.7 ± 1.3 vs 54.6 ± 1.4; P < 0.05, n = 50 crypts) (Fig. 6c,d).

Immunostaining for PCNA in ileal sections showed its expression along the crypt except its lower pole. Irradiation resulted in a significant decrease in PCNA expression in both the crypt and in the lower regions of the villi (31.1 ± 0.8 vs 26.6 ± 0.6; P < 0.001, n = 50 crypts). Treatment using AA-ORS showed significant increase in 5 Gy mice (26.6 ± 0.6 vs 34.2 ± 0.5; P < 0.001, n = 50 crypts) and not in 0 Gy irradiated mice (Fig. 6e,f).

## Discussion

Gastrointestinal toxicity is frequently observed secondary to accidental or therapeutic radiation exposure. Radiation exposure affects intestinal epithelial cells undergoing rapid mitosis in submucosal crypts. In therapeutic radiation exposure gastrointestinal toxicity is quite often becomes a dose-limiting factor for treatment and can affect patients’ quality of life. Therapeutic compounds and supportive care are often used to minimize toxicity, but these approaches are not fully effective.

There has been a growing interest in developing mitigation agents for short-term and long-term GI toxicity in cancer patients and victims of radiation disasters^7^,^8^,^27^,^28^. There are only two FDA approved agents; Neupogen^®^ and Pegfilgrastim (Granulocyte-colony stimulating factor) are the two FDA-approved medical countermeasure that is currently available to treat radiation syndrome. Both work to increase survival in patients exposed to myelosuppressive doses of radiation. However, there are no agents that specifically address gastrointestinal toxicity. Treatment of GI toxicity is mostly symptomatic, with antidiarrheal used to prevent fluid loss, smectite to absorb bile salts, opioids to relieve stomach or rectal pain, steroids to relieve inflammation and in extreme cases parenteral feeding to correct malabsorption of nutrients and electrolytes. Other agents that could potentially be used for mitigating GI toxicity are; 1) Statin and/or angiotensin-converting enzyme, this agent has been found effective when used during radical pelvic radiotherapy works by its anti-inflammatory, antifibrotic and antithrombotic actions; 2) Antioxidants such as vitamin E and/or selenium; 3) Teduglutide, a glucagon-like peptide-2 analogue that has to be given prior to radiation; 4) Sucralfate, a highly sulphated polyanionic disaccharide helps in epithelial healing, but has not been shown to be useful in radiation-induced GI toxicity; 5) Nitroxides such as hydroxylamines (tempol), works by its antioxidant properties; 6) Dithiolthione (Oltipraz), works by increasing sulfhydryl in cells; 7) Isoflavone (genistein), a tyrosine kinase inhibitor and antioxidant; 8) Cox-inhibitors (celecoxib, aspirin), work by increasing Cox2 activity and prostaglandin synthesis; and 8) Probiotics, a preparation containing viable and well defined microorganisms in large numbers to alter hosts microflora and may have some effect on radiation-induced GI toxicity^7^,^29^.

The amino acids based oral rehydration solution used in this study works by correcting the functional changes that happened at the GI mucosa following radiation. The amino acids were selected to counter the increased paracellular permeability, increased Cl^−^ secretion and decreased absorption of electrolytes following radiation. Since, the AA-ORS corrects functional alteration in the GI mucosa, its action is thought to be upstream of the current agents in the pipeline. The amino acids used in the formulation are classified as food, and the agent could be administered with other therapeutic agents. We recently found that electrolytes, glucose, and some amino acids are poorly absorbed in the GI tract following irradiation. In addition, we observed that glucose and some amino acids can stimulate electrogenic Cl^−^ secretion in addition to Na^+^ absorption and can increase paracellular permeability, which further complicates radiation-induced diarrhea and increased gut permeability^20^,^30^. Increased paracellular permeability is known to increase translocation of antigenic substances from the gut lumen into the systemic compartment, causing an increase in pro-inflammatory cytokines^20^.

We found that increased weight gain and survival could be secondary to increased crypt number and villus height that then increased the surface area of absorption. We have demonstrated that crypt number and villus height increased with AA-ORS treatment beginning 6 days after irradiation. Using the single-hit, multi-target model for crypt survival, we found that the number of crypt progenitor units per ileal circumference (N) increased significantly (P < 0.001) without a change in D_0_ (4.8 ± 0.1 Gy) (Fig. 1**a**). The D_q_ values improved the equivalent to an increased radiation tolerance of 1.7 Gy with AA-ORS treatment, indicating improved crypt survival. The crypt survival studies suggested an increase in progenitor units or stem cells per crypt. Thus, we examined the effect of irradiation and AA-ORS on stem cell number using antibodies specific to intestinal stem cell markers and migration of the daughter cells into the villus secondary to proliferation by EdU incorporation^15^,^16^,^17^. At least three distinct crypt cell types are postulated to represent intestinal stem cells (ISC)^15^. Each member of the population has distinct proliferation kinetics and sensitivities to radiation; therefore, each is thought to serve a unique function^31^. They are believed to dynamically switch from one type to the other in response to inhibitory and stimulatory signals caused by cytokines, hormones, or growth factors^32^. In contrast, slow-cycling intestinal epithelial stem cells (IESC) [label-retaining cells (LRC)] at the “+4 crypt position” contribute to homeostatic regenerative capacity, particularly during recovery from injury^33^. These LRC express various markers, such as Bmi1, HopX, Lrig1, and/or Dclk1, and can change to rapidly cycling IESCs in response to injury^34^. Lgr5 can mark both cells, whereas Bmi1 and HopX were reported to preferentially mark +4 cells^15^. Lgr5^+^ ISC are necessary for intestinal regeneration following radiation injury^35^. Lgr5^−^ and Bmi1 are thought to be reserve cells that mount regenerative response following injury or radiation-induced damage. Studies have shown that the loss of Lgr5^+^ cells is tolerated due to activation of the Bmi1-expressing stem cell pool^15^,^35^.

We found that 5 Gy resulted in a significant decrease in Lgr5 transcript and protein levels without much change in Bmi1 levels when compared to 0 Gy. This study supports previous observations that Lgr5^+^ stem cells, unlike Bmi1^+^ stem cells, is more sensitive to radiation and toxic injury, which decreases their population in the crypt^33^,^34^. We found a significant enhancement in the Lgr5 mRNA and protein levels without much change in Bmi1 with AA-ORS treatment, suggesting increased Lgr5^+^ stem cells. Our results also showed that the length covered by the migrating cells was significantly greater in intestinal sections from AA-ORS-treated irradiated mice when compared with saline-treated mice. However, the study does not determine if the increased Lgr5^+^ cells with treatment are secondary to increased survival of Lgr5^+^ cells or by dynamically switching from a Bmi1-expressing stem cell pool to form Lgr5^+^ cells. Since transcription is halted in cells undergoing apoptosis, it is plausible that increased cell survival preferentially elevates short-lived transcripts, such as *Lgr5*, over the long-lived transcripts. Bmi1 protein levels in intestinal tissues did not change in response to radiation or AA-ORS, thereby suggesting that the reserve populations of ISC are not affected at the radiation dose studied. Also, the increase in Lgr5 protein levels with AA-ORS supports our observation in the crypt count study that AA-ORS treatment following increasing doses of radiation, when fit in as a single-hit, multi-target model, leads to an increase in ISC number.

In an effort to understand the potential mechanism by which the amino acids activate the stem cell number or its proliferation, we studied the role of Erk, a protein known to communicate cell surface signals to the nucleus for mediating the transcriptional and translational changes necessary to bring about proliferation. In a resting condition, Erk is anchored to the cytoplasm by the microtubule network or by phosphatases^36^. Erk1 and Erk2 are 44-kDa and 42-kDa proteins that are an important subfamily of mitogen-activated protein kinases that control a broad range of cellular activities and physiological processes, including cell proliferation and differentiation by down-regulating pro-apoptotic molecules and upregulating anti-apoptotic molecules^37^. Activation of Mek1/2 leads to the phosphorylation of threonine on tyrosine residues of Erk1 and Erk2^38^,^39^. Upon stimulation, Erk1/2 becomes phosphorylated on threonine and tyrosine residues, and the latter results in the dissociation of Erk1/2 from Mek1/2. Erk1/2 then translocates to the nucleus. Nuclear translocation persists during the entire G1 phase and can be reversed by removing the mitogenic stimulus^40^. Since AA-ORS increased p-Erk, this study suggest that the amino acids help maintain the mitogenic stimulus until late G1 for successful S-phase entry^41^.

Akt, also known as protein kinase B, is a serine/threonine-specific protein kinase that plays a role in cell proliferation and survival and inhibits apoptosis and metabolism. Phosphorylation of Akt at S473 and T308 activates Akt^42^,^43^,^44^. Like p-Erk, Akt is also known to play a role in the cell cycle. Akt has been shown to overcome the cell cycle arrest in G1 and G2 phases^45^,^46^. Akt could also promote growth factor-mediated cell survival. A variety of studies have documented the key role of the Akt pathway in preventing apoptotic cell death^47^. PCNA, a distinctive protein linked to DNA replication and therefore used as a marker for proliferation was measured with AA-ORS or saline treatment. AA-ORS increased PCNA in 0 Gy and 5 Gy irradiated mice, but not in saline treated mice. Increase in PCNA is an early indication for small intestinal epithelial proliferation. Together these studies suggest enhanced proliferation with treatment using AA-ORS.

Another executioner or effector of apoptosis is caspase-3, as cleaving of protein substrates within the cell leads to morphological changes associated with apoptosis, including DNA degradation and chromatin condensation, and membrane blebbing to trigger the apoptotic process^48^. This inactive pro-enzyme is activated by proteolytic cleavage^49^,^50^. Our study showed that radiation increased caspase-3 and that AA-ORS treatment decreased cleaved caspase-3 in the villus epithelial cells of 0 Gy and 5 Gy mice. Bcl-2, a downstream target for Erk1/2, is known to inhibit Bax in the intrinsic pro-apoptotic pathway. Increased Bcl-2 protein levels with AA-ORS suggest a protective mechanism to prevent apoptosis. However, increased protein levels of Bcl-2 in tissues from irradiated mice may suggest a radio-protective mechanism. Similar increase in Bcl-2 protein levels following irradiation has been reported and agree with our findings^51^. However, Bax protein failed to show significant changes with radiation or with treatment, suggesting AA-ORS effect on apoptosis at a step upstream to Bax^26^. Increased p-Akt in AA-ORS-treated mice suggests its action may be by activation of proliferation or inhibiting apoptosis (Fig. 5). Together with the effects seen on caspase-3 and Bcl-2, these results could explain the pro-survival effect and increased proliferation observed with AA-ORS treatment. However, further studies will be essential to characterize the mechanisms by which AA-ORS activates Erk1/2 and Akt, PCNA caspase-3, Bcl-2 or Bax.

**Figure 7:**
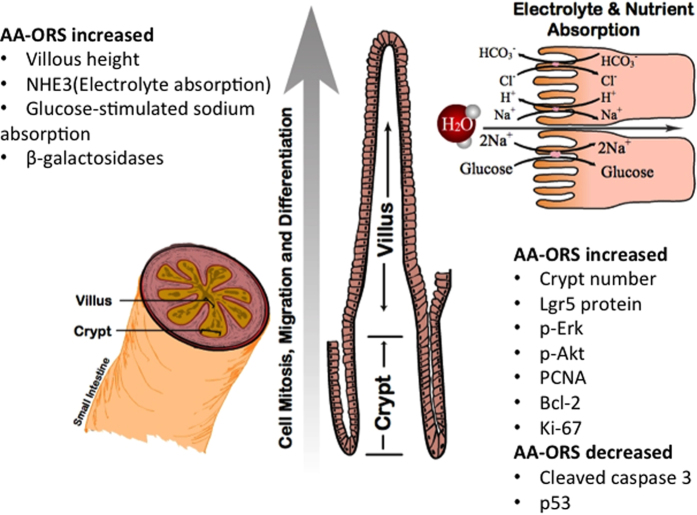
Schematic figure of small intestinal villus and enterocytes: AA-ORS treatment increases rapidly dividing stem cells that are Lgr5^+^, as well as proliferation markers p-Erk, p-Akt and PCNA. The treatment also increases cleaved caspase-3, p53 and Bcl-2. The AA-ORS treatment increases villus height, increased expression of NHE3, SGLT1 and β-galactosidases thereby increasing electrolyte absorption, sodium-coupled glucose absorption, and breakdown of disaccharides at the brush border membrane, respectively. A cartoon of the enterocyte on the top right shows the functional improvement in NHE3 mediated Na^+^ absorption and glucose-coupled sodium transport with AA-ORS.

Since Akt could also play prominent roles in malignant transformation^52^, we studied the role of p53, a known tumor suppressor protein, with AA-ORS. Changes in the p53 protein may suggest that AA-ORS has tumor-suppression effects. Mutations in the p53 tumor-suppressor^53^ gene are the most frequently observed genetic lesions in human cancers. Mice homozygous for the null allele appear normal but are prone to the spontaneous development of a variety of tumors^54^. p53 has also been shown to play an important role in the radiation response; indeed, the level of p53 accumulation in response to irradiation primarily results from the intensity of DNA damage^55^. Studies have shown that stem cell loss plays an important role in radiation-induced acute intestinal injury and lethality and is regulated by the p53 pathway and its transcriptional targets PUMA and p21^27^,^28^,^56^. PUMA-dependent apoptosis quickly reduces ISC and its progenitors in hours following high-dose irradiation, and deficiency of PUMA leads to improved animal survival and crypt regeneration by enhancing p21-dependent DNA repair and is crucial for radiation-induced intestinal damage^57^,^58^. Together with Lgr5, p-Erk, and p-Akt, the changes in cleaved caspase-3 suggest that AA-ORS increased villus height in intestinal tissues from non-irradiated and irradiated mice not only through proliferation but also through decreased apoptosis and increased cell survival. To assess if the villus epithelial cells resulting from increased proliferation and decreased apoptosis are mature, differentiated, and functionally active, we measured Na^+^ absorptive capacity and glucose-stimulated Na^+^ absorption. Both NHE3, the predominant transporter of Na^+^ absorption in the small intestine, and SGLT1, the transporter for sodium-coupled glucose absorption, were only found in mature and differentiated villus cells; they had increased function (Figs 3 and 4) as well as increased mRNA and protein levels. These studies suggest that AA-ORS treatment following irradiation increased electrolyte and glucose absorption (Figs 3, and 7).

An amino acid formulation that increases villus height has important implications for disease conditions characterized by a decrease in villus height that are outside of radiation or chemotherapy-induced toxicity, such as Crohn’s disease, celiac disease, malnutrition, and environmental enteropathy. This study signifies how a systematic selection of certain nutrients based on their beneficial effect on GI function helped to improve *in situ* intestinal stem cell proliferation, maturation, and differentiation, leading to functionally active long villus epithelial cells whose function and height were initially compromised by irradiation (Fig. 7). The study also supports Brain J. Leibowitz *et al.’s* observation that bone marrow derived stem cells have no significant role in the repopulation of intestinal mucosa following high dose radiation^14^. Future studies should seek to determine the mechanisms by which these amino acids increase the stem cell population, increase their proliferation, and decrease apoptosis and also to rule out malignant transformation. Our work highlights the importance of careful selection of different nutrients or individual amino acids to affect various stem cell populations, including hematopoietic stem cells.

## Methods

### Animal model

Eight-week-old, male, NIH Swiss mice were fed a normal diet and housed at 4 mice per cage. The mice were irradiated using a Gammacell 40 Exactor Low-Dose Research Irradiator (Best Theratronics, Ottawa, Ontario) housing two cesium-137 sources in a parallel and opposed geometry to deliver isotropic irradiation with dose uniformity within ± 3%. Most of the models for mitigation of radiation involve a combination of mucosal depilation and nutritional death within 7 days (mouse) and are preceded with vomiting (humans) within hours to days and maximum epithelial depilation at 3.5 days (mouse). All these models involve total body irradiation (TBI), sometimes with partial femur protection or a marrow transplant^4^,^59^. Selection of a TBI model was to avoid any circulating progenitor cells from migrating into tissues and thus, focuses on *in situ* repopulation. In this study, mice received a single fraction of TBI at a dose rate of 0.9 Gy/minute. Mice were secured in the middle of the irradiation chamber with a plastic jig that allowed 5 mice to be irradiated simultaneously. Mice treated with the formulation were given AA-ORS by gastric gavage once daily (0.3 mL/mouse). Control groups were given normal saline. The amino acid formulation was given as a supportive therapy and was not part of replacement therapy. Absorption of Na^+^ as measured using ^22^Na flux in tissues mounted in Ussing chambers showed better net Na^+^ absorption in mice fasted for 8-hour prior to gastric gavage when compared to animals that had unrestricted access to food (3.4 ± 0.5 μeq. h^−1^. cm^−2^ vs 1.8 ± 0.4 μeq. h^−1^. cm^−2^, P < 0.01; n = 8 tissues from different mice). Therefore, in all further experiments mice were fasted for 8 hours prior to gavage. Animals were humanely euthanized through CO_2_ inhalation followed by cervical dislocation (per the AVMA Guidelines for the Euthanasia of Animals) 6 days after irradiation when peak anion secretion occurs. Toxicity was predominantly due to acute GI syndrome and only minimally perturbed by bone marrow syndrome^19^. Following exsanguination, the ileal mucosa was obtained as previously described^19^,^21^. Glucose-stimulated and amino acids-stimulated currents were higher in ileum when compared to jejunum (4.8 ± 0.5 μeq. h^−1^. cm^−2^ vs 1.1 ± 0.4 μeq. h^−1^. cm^−2^, P < 0.001; n = 8 tissues from different mice) and therefore ileum was selected for all the studies. These observations were similar to past observations where ileum was shown to have greater amino acid absorptive capacity than jejunum^60^. All experiments were approved by the University of Florida Institutional Animal Care and Use Committee (IACUC) and carried out in accordance with IACUC protocol #3875.

### Crypt count and villus length measurements

Paraffin sections (5 μm) were obtained from intestinal segments oriented such that the sections were cut perpendicular to the long axis of the intestine. For determination of the cell survival curve parameters, the crypt counts were normalized and analyzed using the classical method^61^.

### Cell proliferation and crypt-to-villus migration assay

Incorporation of 5-ethynyl-2′-deoxyuridine (EdU, a thymidine analogue) into cellular DNA and the subsequent reaction of the EdU with a fluorescent azide in a copper-catalyzed reaction were used to study cell proliferation in the crypt cell region. Mice were injected (i.p.) with 0.5 mg of EdU in 150 ml of PBS (16.7 mg/Kg) to assess mitotic activity in the crypt cells (these studies reveal S-phase in the crypts) and euthanized at 24, 48, and 72 hours after injection. Paraffin sections from the mouse ileum were prepared, and incorporated EdU (Thermo Fisher Scientific Cat #A10044) was visualized following the manufacturer’s instructions (Alexa 647 imaging kit, Cat #C10340). The sections were then mounted in fluorescent mounting media with DAPI (VectaShield, Cat #h−1200). Figure 1 shows positively stained enterocytes migrating from the base of the crypt to the tip of the villus; the resulting percentage was plotted against the induction time.

### Flux studies for Na^+^ and Cl^−^ absorption

Stripped ileal sheets were mounted in between 2 halves of an Ussing chamber with 0.3 cm^2^ of exposed surface area (P2304, Physiologic Instruments, San Diego, CA, USA). Tissues were bathed in Ringer solution. The Ringer solution contained (mmol.L^−1^) Na^+^ 140, Cl^−^ 119.8, K^+^ 5.2, HPO_4_^−^ 2.4, H_2_PO_4_^−^ 0.4, Mg^2+^ 1.2, Ca^2+^ 1.2, and HCO_3_^−^ 25. The Ringer solution was bubbled with 95% O_2_ and 5% CO_2_ bilaterally, and was maintained at 37 °C. After the tissues were allowed to stabilize for 45 minutes, the basal short-circuit current (*I*_*sc*_), expressed as μeq·h^–1^·cm^–2^, and conductance (*G*), expressed as mS. cm^−2^, were recorded using a computer-controlled voltage/current clamp device (VCC MC-8, Physiologic Instruments), as previously described^19^,^21^. For flux studies, radioisotopes of Na^+^ (^22^Na) and Cl^−^ (^36^Cl) were used to study Na^+^ and Cl^−^ fluxes across the ileal mucosa, as previously described^19^,^62^. ^22^Na activity was measured using a gamma counter (Wizard 2, 2480 Automatic Gamma Counter, Perkin Elmer, USA), while ^36^Cl was measured using a liquid scintillation counter (LS 6500 Multipurpose Scintillation Counter, Beckman Coulter, Inc., Brea, CA, USA). Net flux (*J*_net_) is calculated from mucosa to serosa and serosa to mucosa unidirectional fluxes determined from separate tissues, paired based on similar conductance (<5% difference) and calculated using the formula *J*_net_ = *J*_ms_ − *J*_sm_.

### Real-time quantitative polymerase chain reaction (PCR)

RNA from intestinal tissue samples of 0 Gy and 5 Gy mice was extracted using the TRIZOL method. C-DNA was prepared with a c-DNA kit (iScript™ Select cDNA Synthesis Kit, Bio-Rad, Hercules, CA) as per the manufacturers protocol; Quantitative PCR was performed using specific oligonucleotide primers for NHE3, Lgr5, SGLT1, Erk, Akt Bmi1 and caspase-3. c-DNA (2 μL) was added to 18 μL of SyBr green mixture in CFX Connect Real-time System Cycler (Bio-Rad) for qPCR. One cycle consisted of 30 sec at 94 °C for denaturation, 60 sec for annealing, and 90 sec at 72 °C for extension. CFX Manager™ Software (Bio-Rad) was used for analysis. Standardization of the mRNA used the delta-delta Ct (DDCt) method. Briefly, DCt = Ct (target gene-treated) – Ct (ref gene-treated) and DCt = Ct (target gene-control) – Ct (ref gene – control). Therefore, DDCt = DCt (treated) –Ct (control). Fold change was calculated from the formula 2^(−DDCt)^.

### Western Blot

Total cell lysate from nonirradiated and irradiated AA-ORS-treated or saline-treated mice was prepared in ice-cold RIPA buffer [50 mmol/L Tris-HCl (pH 7.4), 150 mmol/L NaCl, 1% IGEPAL, 1 mmol/L EDTA, 0.25% sodium deoxycholate, 1 mmol/L sodium fluoride, 1 mmol/L sodium orthovanadate, 0.5 mmol/L PMSF, 10 μg/mL aprotinin, 10 μg/mL leupeptin]. The protein concentration in each extract was determined by BCA assay (Sigma, St. Louis, MO). Cell extracts were subjected to sodium dodecyl sulfate polyacrylamide gel electrophoresis (SDS-PAGE); proteins were transferred to polyvinylidene difluoride (PVDF) membranes and probed with primary antibodies that detect Lgr5, SGLT1, Bmi1, caspase-3, p-ERK, and total ERK. Signals were detected with Odyssey CLX from LI-COR. Reversible Coomassie blue stain (Fisher Cat #20278) was used, according to the manufacturer’s instructions, to check equal loading of gels. The abundance of the protein of interest was normalized to the total protein density in each lane of Commassie blue stained gels. This technique minimized variations associated with comparing protein density to a single protein.

Immunohistochemical identification of the nuclear proteins PCNA and Ki-67, and the stem cell-specific membrane protein Lgr5 was performed using polyclonal rabbit anti-mouse GPCR (Lgr5) (Abcam Cat# ab75732), Ki-67 (Abcam Cat# ab15580) and PCNA (Abcam Cat# ab18197) antibodies. A rabbit specific ABC detection kit (Abcam Cat# ab64261) was used to visualize the expression of the protein according to the manufacture’s instruction. Briefly, formalin-fixed, paraffin-embedded full-thickness ileum samples were cut into 4 μm thick cross-sections, mounted on Superfrost Plus glass slides, de-paraffinized and rehydrated. For antigen retrieval heat pretreatment was applied using a pressure cooker (125 °C for 30 sec, and 90 °C for 10 sec) and retrieval buffer; Deloaker RTU Buffer (Biocare Medical Cat #RV1000MMRTU) at pH 6.0. After quenching endogenous peroxidase and blocking nonspecific bindings, sections were incubated with primary antibody diluted in PBS (Lgr5 – 1:100, Ki-67 – 1:1000, PCNA - 1:4000, for 2 hours; 15 min; and 2 hours respectively at room temperature). PBS was used as a negative control. Tissues were then incubated with biotinylated goat anti-rabbit secondary antibody for 10 minutes. After incubation with streptavidin peroxidase, the desired stain intensity was obtained with Di-amino-benzidine by visualizing under the microscope. Sections were counterstained with Mayer’s hematoxylin (Electron Microscopic Sciences (EMS) Cat #26043-05), dehydrated and mounted in Permount mounting medium (Fisher Scientific Cat #SP15-100). Slides were evaluated by light microscopy using a 20× objective for PCNA and Ki-67, and a 40× objective with oil immersion for Lgr5. The number of positive-brown cells were counted from 50 crypts per group and analyzed.

#### Statistics

Results are presented as mean ± standard error of mean (S.E.M.). Statistical analysis was performed in 2 steps: 1) overall difference was tested using analysis of variance (ANOVA) (or its non-parametric equivalent Kruskal-Wallis); and 2) Bonferroni-adjusted *P*-values were computed for all pair-wise comparisons.

## Additional Information

**How to cite this article**: Yin, L. *et al*. An amino acid-based oral rehydration solution (AA-ORS) enhanced intestinal epithelial proliferation in mice exposed to radiation. *Sci. Rep.* **6**, 37220; doi: 10.1038/srep37220 (2016).

**Publisher's note:** Springer Nature remains neutral with regard to jurisdictional claims in published maps and institutional affiliations.

## Supplementary Material

Supplementary Figures
