# Supplementary material for: An amino acid-based oral rehydration solution (AA-ORS) enhanced intestinal epithelial proliferation in mice exposed to radiation
Source: Sci Rep. 2016 Nov 23;6:37220. doi: 10.1038/srep37220 (PMC5120277; doi:10.1038/srep37220)
Supplement: Supplementary Figures [file srep37220-s1.pdf]

An amino acid-based oral rehydration solution (AA-ORS) enhanced intestinal epithelial proliferation in mice exposed to radiation

Liangjie Yin,[1] Reshu Gupta[1], Lauren Vaught[1], Astrid Grosche[1], Paul Okunieff,[1]

Sadasivan Vidyasagar[1*]

[1]Department of Radiation Oncology, University of Florida Health Cancer Center, Cancer and Genetics Research Complex, 2033 Mowry Road, Box 103633, Gainesville, FL 32610

[*]Corresponding author:

Sadasivan Vidyasagar, PhD

Department of Radiation Oncology

University of Florida Health Cancer Center

Cancer and Genomic Research Complex

2033 Mowry Rd.

Box 103633

Gainesville, FL 32610

E-mail**:** vidyasagar@ufl.edu

Telephone: (352) 294-5061; Fax: (352) 265-7045

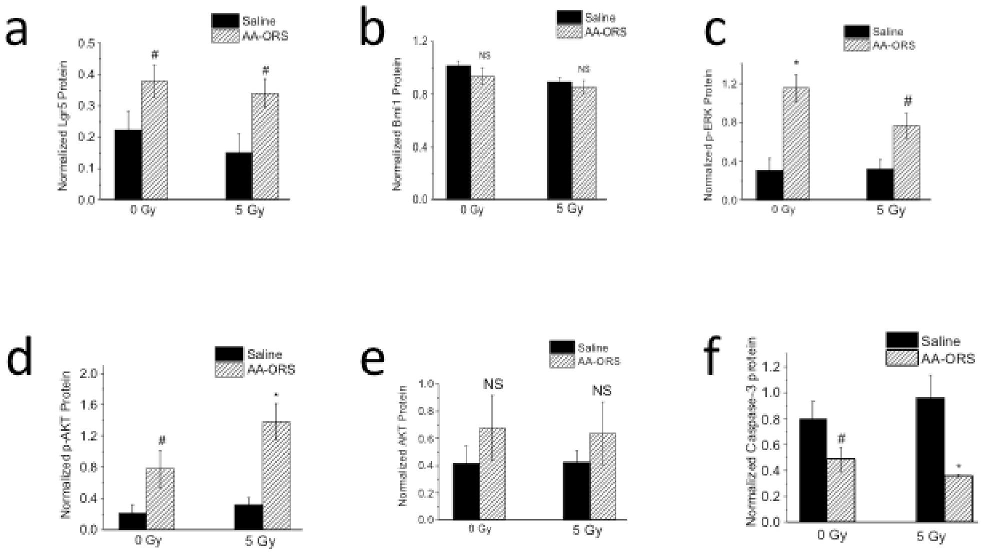

Supplementary Figure 1: Graph showing protein density for Lgr5, Bmi1, p-ERK, p-AKT, AKT and Caspase-3. Protein density was normalized to coomassie blue. Mice treated with saline are shown as black bars and AA-ORS treated are shown as hatched bars following 0 or 5 Gy irradiation. (Values are means ± SEM from n = 4 different mice repeated in triplicate. # *P* < 0.05 compared with saline control).

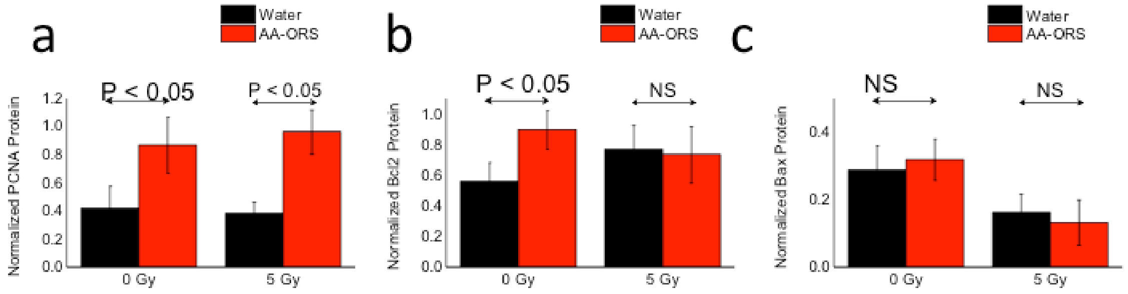

Supplementary Figure 2: Graph showing protein density for PCNA, Bcl-2, Bax. Protein density was normalized to coomassie blue. Mice treated with saline are shown as black bars and AA-ORS treated are shown as red bars following 0 or 5 Gy irradiation. (Values are means ± SEM from n = 4 different mice repeated in triplicate. # $P < 0.05$ compared with saline control).
